# Comparison of IgG4 with inflammatory cytokines (IL-1, IL-6 and TNFα) in rheumatoid arthritis

**DOI:** 10.3389/fimmu.2025.1607074

**Published:** 2025-07-07

**Authors:** Rajalingham Sakthiswary, Syahrul Shaharir, Asrul Abdul Wahab, Veshaaliini Uma Rajeswaran

**Affiliations:** Faculty of medicine, Universiti Kebangsaan Malaysia, Cheras, Malaysia

**Keywords:** rheumatoid arthritis, immunoglobulin G, disease activity, cytokine, joint erosions

## Abstract

**Background:**

Rheumatoid arthritis (RA) is an autoimmune disorder characterized by chronic joint inflammation driven by a complex interplay of autoantibodies, cytokines, and chemokines. While the role of proinflammatory cytokines, such as interleukin-1(IL-1), interleukin-6(IL-6), and tumor necrosis factor-α (TNF-α), in the pathogenesis of RA has been well-established, the contribution of the immunoglobulin G subclass IgG4 remains a topic of ongoing investigation. This cross sectional study aim was to compare the levels of IgG4 and these key inflammatory cytokines in Malaysian patients with RA.

**Methods:**

The study enrolled a total of 194 RA patients. All subjects were tested for their serum IgG4, IL-1, IL-6 and TNF-α levels. Besides, subjects were assessed for their disease activity based on DAS28, functional disability based on HAQ-DI (Health Assessment Questionnaire-Disability Index) and the severity of the radiographic joint erosions by using the Modified Sharp Score (MSS).

**Results:**

Correlation analysis revealed a moderate positive association between IgG4 and IL-6 levels (r=0.348, p=0.001), but there were no significant correlations with IL-1 and TNFα levels. IgG4 levels showed a significant positive correlation with DAS28, MSS, and HAQ-DI. All 3 cytokine levels had significant relationships with the DAS28 scores, but for the MSS, only TNF-α (p=0.024) and IL-6 (p=0.016) demonstrated significant associations. None of the cytokines correlated significantly with the HAQ-DI scores.

**Conclusion:**

The positive correlation between IgG4 and IL-6 levels underscores the possibility for IL-6-driven pathways to influence IgG4 production. Serum IgG4 was associated with more clinical aspects of RA compared to the classical inflammatory cytokines.

## Introduction

Rheumatoid arthritis (RA) is a systemic autoimmune disease that primarily affects the synovial joints. In RA, the activation of the immune system occurs in response to the complex interplay of autoantibodies, cytokines and chemokines. Cytokine-mediated pathways are important in the development of RA. A high production of proinflammatory cytokines and the deregulation of anti-inflammatory cytokines promote the induction of autoimmunity by activating signaling pathways related to chronic joint inflammation (1). Many cytokines have been identified over the years as the main culprits in the pathogenesis of this disease. Nevertheless, there is still controversy regarding the contribution of various autoantibodies in RA.

The role of IgG4 in autoimmune diseases remains elusive. IgG4 is generally considered to be non-inflammatory due to its unique structure. However, in IgG4-related disease, it plays a pathogenic role through fibrinogenesis and inflammatory processes. IgG4 has been implicated in autoimmune diseases such as bullous pemphigoid, idiopathic membranous glomerulonephritis, myasthenia gravis, Sjogren syndrome and, RA (2).

IgG4 may mimic rheumatoid factor (RF) activity by interacting with other immunoglobulins G and may bind with soluble antigen to form immune complexes (3, 4). These immune complexes lead to complement activation, cellular cytotoxicity, opsonization, phagocytosis and inflammation. Zack et al. found that in RA, RF IgG is predominantly the IgG4 subclass (3). It is assumed that in active RA, there is a boost in the production of IgG4 through complex autoimmune mechanisms. Engelman et al. and Carbone et al. demonstrated a significant decrease in IgG4 anti-citrullinated peptide antibodies in responders to treatment (5, 6).

IgG4 levels are significantly elevated in patients with RA compared to the healthy individuals. Understanding IgG4 role and immunopathology is a key step forward towards the development of novel, IgG4 targeted therapies. IgG4 levels vary considerably in the general population and may range from 0.01g/L to 1.4g/L (7). Under normal circumstances, IgG4 is produced after chronic exposure to antigen or allergen. It is usually induced by a class switch from IgE to IgG4. IgG4 then competes with antibodies of other classes and blocks the epitope to suppress the effector function of the competing antibody. IgG4 autoantibodies exert their effects by direct interference with their target antigens and this amplifies the disease activity (8–10).

Synthesis of IgG4 *in vitro* was regulated by certain cytokines such as IL-10 and IL-6 (11). Serum IL-10 was also reported to be higher in RA patients compared to healthy controls (12). The role of IL-10 in IgG4 elevation in RA remains vague and cannot be explained theoretically. However, the role of IL-6 in this regard is clearer.

Immune phenotypes, including cytokine levels and autoantibodies, vary according to ethnicity due to the influence of the exposure to endemic pathogens and the genetic composition. In our previous study, we found that serum IgG4 had significant positive correlations with disease activity, ESR, CRP, joint damage, and functional disability in RA (13). Hence, this exploratory study is an extension of our previous work to evaluate the correlation between the serum cytokines and IgG4 levels in Malaysian RA patients.

## Methodology

### Participants

This study was conducted in a Malaysian tertiary hospital. Investigators identified subjects using the outpatient rheumatology clinic patient lists. RA patients were recruited using convenient sampling. The investigators filled the data collection forms by reviewing the electronic medical records or by interviewing the subjects during the scheduled clinic visits. Data collection occurred between June 2023 and June 2024. This study is an extension of our previous research which was published elsewhere (13). The study was approved by the Research Ethics Committee of the institution (reference FRGS/1/2021/SKK08/UKM/02/1). Pregnant RA patients and those with active infections or malignancies were ineligible.

### Data collection

A standardized form was completed by the investigators. It ascertained information on the following: demographic data (age, ethnicity, gender); disease duration, medications, seropositivity, disease activity based on DAS28, response to DMARDS (disease modifying anti-rheumatic drugs) and functional disability based on HAQ-DI (Health Assessment Questionnaire-Disability Index) (14). All subjects had radiographs of their hands assessed for joint erosions. The severity of the radiographic joint erosions was determined by using the Modified Sharp Score (MSS) (15).

### Measurement of IgG4 and cytokines

Serum was collected from all RA patients at enrollment and stored at −80^∘^C. About 3–5 mls of blood samples were taken from the subjects and processed in a centrifugal separator at 3000 rpm for 15 minutes. The serum concentration of IgG4 was tested using a commercially available enzyme-linked immunosorbent assay (ELISA). The concentrations of interleukin-1 (IL-1), interleukin-6 (IL-6) and tumor necrosis factor α (TNFα) were determined using a multiplex cytokine assay.

### Statistical analysis

Statistical significance was set at p <0.05. Statistics were performed using IBM SPSS version 29. Data were described with mean ± standard deviation or number (percentage) unless stated otherwise. Spearman’s rank order correlation test was used to assess the correlation between two variables. The one-way analysis of variance (ANOVA) test was used to compare the means of 3 or more independent groups, whereas the independent T-test compared the means of 2 groups.

## Results

### Characteristics of the study patients and their IgG4 levels

The study enrolled a total of 194 patients diagnosed with RA according to the 2010 ACR/EULAR classification criteria (16). Majority of the subjects were middle-aged women with predominantly seropositive disease. More than one-third of the subjects had moderate disease activity with moderate response to DMARDS. In this cohort, only a small proportion of subjects had high disease activity (5.7%),which explained the high percentage (74.2%) with IgG4 levels below 86mg/dL (**Table 1**).

**Table 1:** Baseline characteristics of study subjects.

Parameter	N = 194
Age (years)	59.27 ± 12.64
Gender, n (%)	
Men	22 (11.3%)
Women	172 (88.7%)
Ethnicity, n (%)	
Malay	104 (53.6%)
Chinese	64 (33%)
Indian	26 (13.4%)
Disease duration(years)	10.25 ± 7.85
Seropositive disease, n (%)	161 (83.0%)
Number of DMARDs, n (%)	
0	6 (3.1%)
1	70 (36.1%)
2	104 (53.6%)
3	13 (6.7%)
Conventional synthetic DMARDs, n (%)	
Methotrexate	119 (61.3%)
Leflunamide	65 (33.5%)
Sulfasalazine	50 (25.8%)
Hydroxychloroquine	63 (32.5%)
Advanced therapy,n (%)	
Janus Kinase inhibitors	16 (8.2%)
Tocilizumab	3 (1.7%)
TNFα inhibitors	2 (1.2%)
CRP (mg/dL)	0.93 ± 1.38
ESR (IU/ml)	46.92 ± 24.69
DAS28	3.25 ± 1.1
Remission, n (%)	53 (27.3%)
Low Disease Activity, n (%)	37 (19.1%)
Moderate Disease Activity, n (%)	93 (47.9%)
High Disease Activity, n (%)	11 (5.7%)
MSS	29.35± 27.45
HAQDI	0.87 ± 1.0
EULAR response Criteria	
None, n (%)	49 (25.3%)
Moderate, n (%)	73 (37.6%)
Good, n (%)	53 (27.3%)
Serum IgG4 levels (mg/dL)	
0 – 86mg/dL, n (%)	144 (74.2%)
>86mg/dL, n (%)	50 (25.8%)

Data is presented as either count (percentages) or mean ± SD.

CRP, C-reactive protein; ESR, erythrocyte sedimentation rate; DAS28, 28 joint-based Disease Activity Score; MSS, modified Sharp score; HAQ-DI, Health Assessment Questionnaire Disability Index.

### IgG4 and cytokines

**Figure 1:**
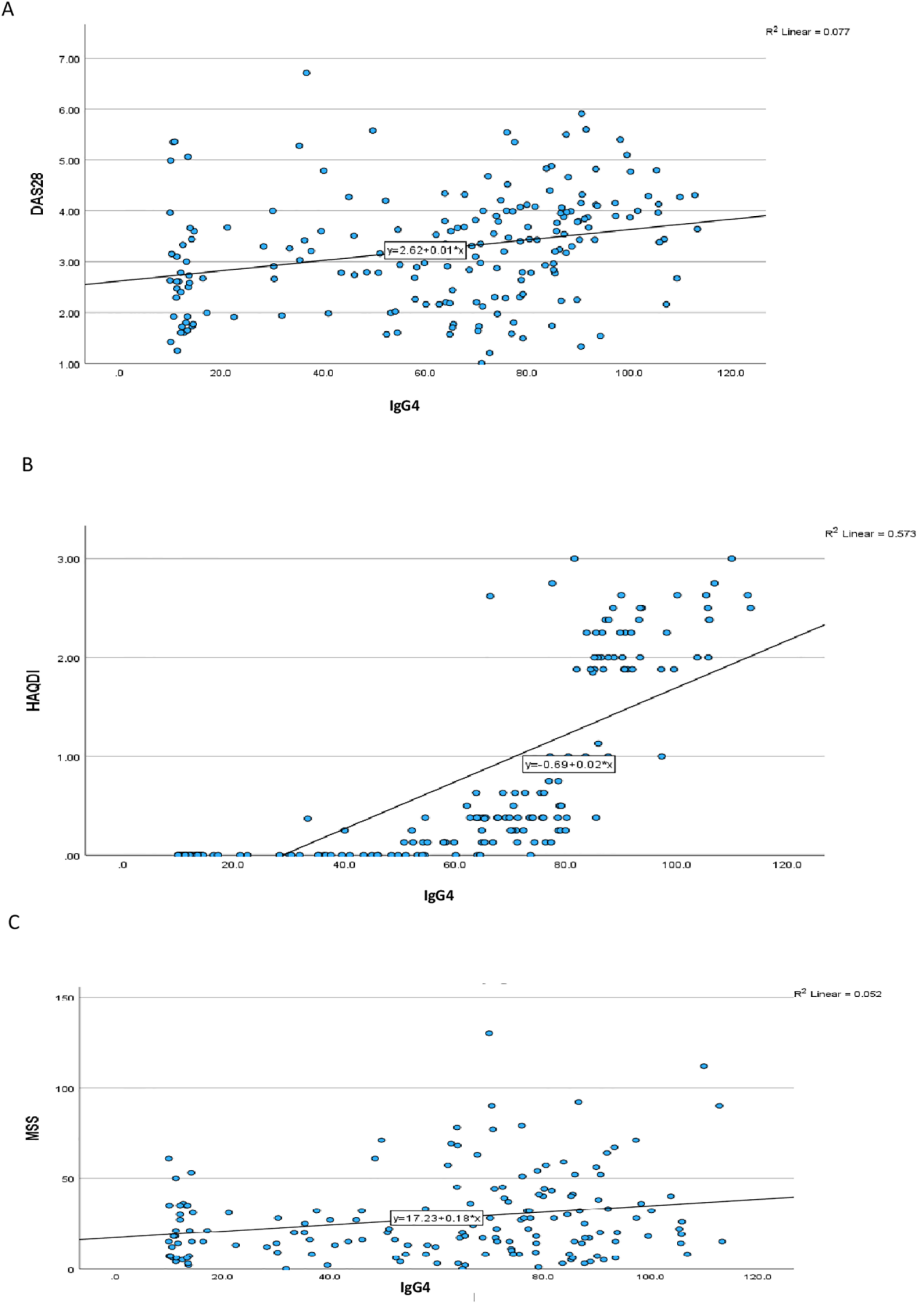
Correlation between IgG4 levels and **(A)** DAS28 **(B)** HAQDI and **(C)** MSS.

Correlation analysis revealed a moderate positive association between IgG4 and IL-6 levels (r=0.348, p=0.001), suggesting a potential link between IgG4 production and IL-6-driven inflammation. This finding is consistent with previous reports indicating that IL-6 can regulate the synthesis of IgG4 *in vitro*. In contrast, the relationships between IgG4 and IL-1 or TNF-α were less pronounced. IgG4 levels showed significant positive correlation with DAS28, MSS and HAQ-DI (**Table 2**) (**Figure 1**). All 3 cytokine levels had significant relationships with the DAS28 scores but for the MSS, only TNF-α (p=0.024) and IL-6 (p=0.016) demonstrated significant associations. None of the cytokines correlated significantly with the HAQ-DI scores.

**Table 2:** Correlation of serum IgG4 with cytokines and disease characteristics.

Parameters	Nilai r	Nilai p
IL-1	0.117	0.104
IL-6	0.348	**0.001**
TNFα	0.081	0.261
ESR (IU/ml)	0.135	0.060
DAS 28	0.278	**<0.001**
MSS	0.237	**0.002**
HAQ-DI	0.769	**0.001**

CRP, C-reactive protein; ESR, erythrocyte sedimentation rate; DAS28, 28 joint-based Disease Activity Score; MSS, modified Sharp score; HAQ-DI, Health Assessment Questionnaire Disability Index.

Statistically significant values are in bold.

### IgG4 and cytokine levels according to disease activity and the presence of joint erosions

**Figure 2:**
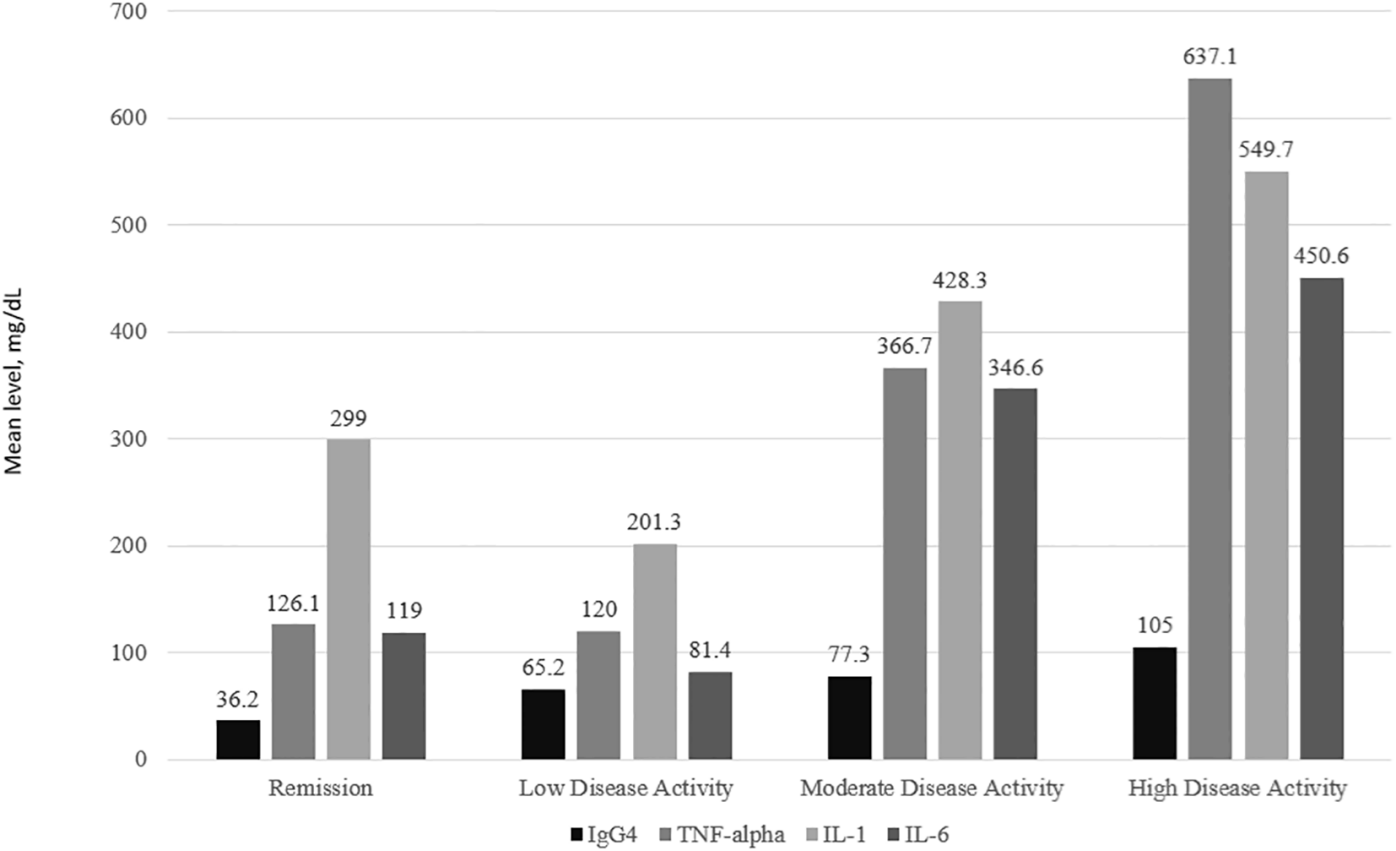
IgG4 and cytokine levels according to disease activity.

**Figure 3:**
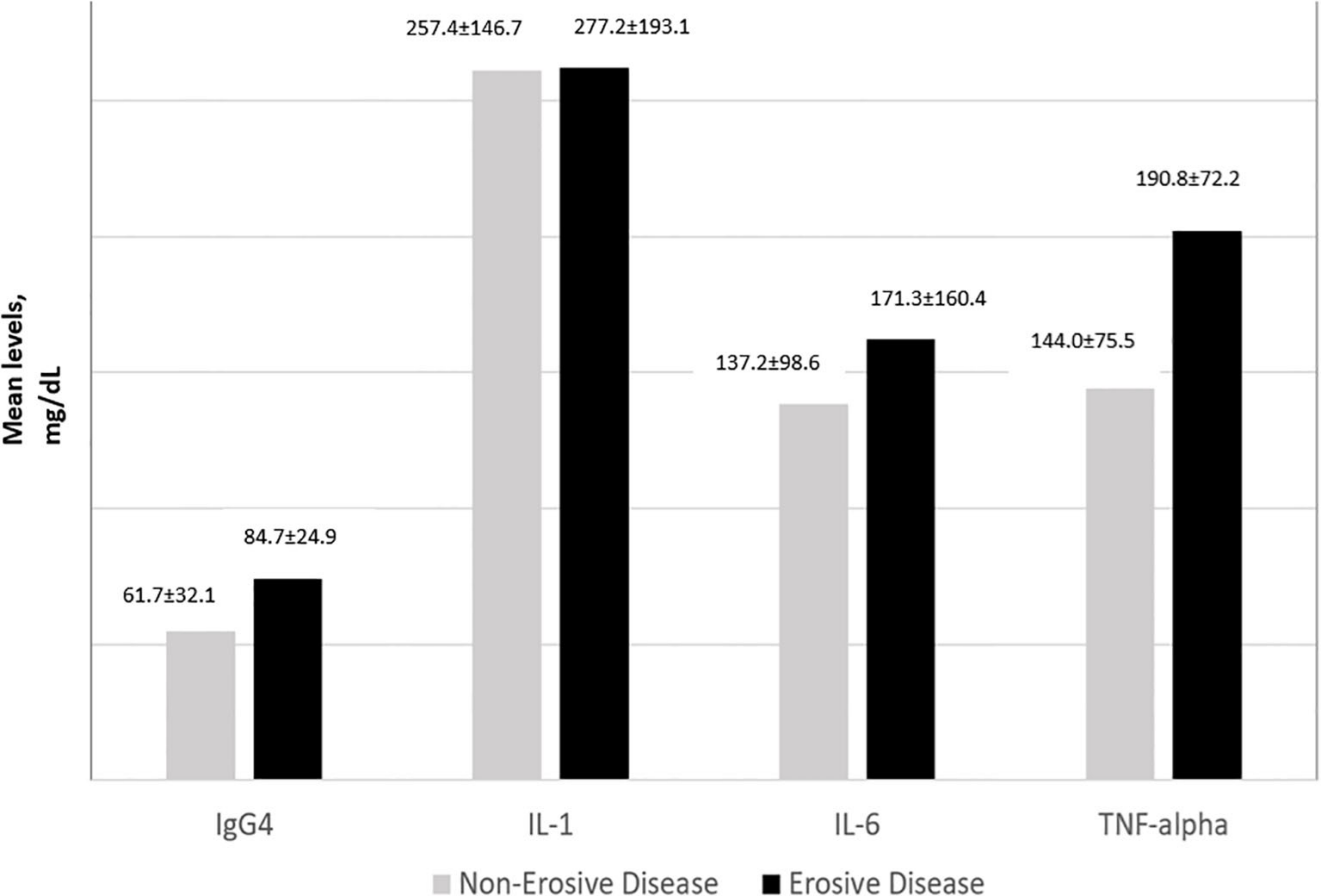
IgG4 and cytokine levels in erosive disease.

IgG4 and all 3 cytokine levels were consistently the highest among subjects with high disease activity followed by moderate disease activity. For IL-1, IL-6 and TNFα, subjects with low disease activity had lower levels of the cytokines compared to those in remission (**Figure 2**). The one-way ANOVA test with the disease activity category as the factor revealed that there was a significant difference in the IgG4(p<0.001), IL-6 (p=0.010), IL-1 (p< 0.001), and TNF-α (p=0.020) levels across the 4 categories of disease activity. Subjects with erosive disease had significantly higher levels of TNF-α(p=0.011), IL-6 (p=0.038), and IgG4 (p<0.001). The mean IL-1 levels were comparable in erosive disease and non-erosive disease (**Figure 3**).

## Discussion

The elevated levels of IgG4 observed in RA patients support the growing body of evidence implicating this immunoglobulin subclass in the pathogenesis of the disease. This study highlighted the significant relationship between serum IgG4 and IL-6 levels in RA patients. The positive correlation between IgG4 and IL-6 levels underscores the potential for IL-6-driven pathways to influence IgG4 production, which may have implications for the development of targeted therapies. IL-6, a principal mediator in inflammatory conditions, promotes the expansion and activation of T cells and the differentiation of B cells. In IgG4-related disease (IgG4-RD), serum IL-6 levels significantly correlated with clinical inflammatory parameters such as serum CRP, hemoglobin, and albumin (17). However, the mechanism of IgG4 elevation seems different between RA and IgG4-RD. The relationship between IL-6 and IgG4 demonstrated in this study might be partly through IL-21 expressed in CD4+ T cells (23), which promotes the differentiation of B cells into antibody-secreting plasma cells (18). In keeping with the above, serum IgG4 levels reduced in 7 of 8 RA patients who received tocilizumab therapy, an anti-IL-6 receptor monoclonal antibody (5).

Although IL-1 and TNFα have been strongly implicated in the pathogenesis of RA, we were unable to demonstrate a significant correlation between the above cytokines and IgG4 levels. A plausible explanation for this finding could be that IL-6 and IgG4 are involved in B-cell driven pathways, whereas IL-1 and TNFα predominantly in T-cell driven pathways (19). Animal studies have suggested that TNFα was an inflammatory mediator, whereas IL-1 was the culprit cytokine in both inflammation and cartilage destruction. As opposed to IL-1, TNFα alone was not destructive, but it could enhance the cartilage destruction and joint erosions by IL-1 (20). Subjects with erosive disease in this study did not have significantly higher levels of IL-1. This was in contrast with the findings of most arthritis studies, which proved that transgenic over-expression of IL-1 led to bone destruction (21).

Unlike IgG4, none of the cytokines had an appreciable relationship with the HAQ-DI scores. Studies on autoantibodies and functional disability have provided mixed results. Anti CCP and RF levels did not correlate with HAQ-DI in a few studies (22, 23). On the contrary, a few studies have demonstrated a link between anti-CCP levels and the severity of functional disability (24, 25).

There were a few limitations in the current study. Firstly, the study was conducted cross-sectionally. Cytokines tend to fluctuate with time. Secondly, IgG glycosylation and the size of the immune complexes play an important role in autoimmune diseases, but these molecules were not included in this study. Thirdly, due to small sample size, it was not possible to perform multivariate analysis on the relationship between IgG4 levels and the variables that correlated significantly.

In conclusion, this study provides a comprehensive comparison of IgG4 and key inflammatory cytokines in patients with RA. The findings highlight the complex interplay between humoral and cytokine-mediated immunity in the pathogenesis of RA, and underscore the need for further investigation into the specific mechanisms by which IgG4 contributes to disease progression.

## Data Availability

The datasets presented in this article are not readily available because of institutional restrictions. Requests to access the datasets should be directed to sakthis5@hotmail.com.
